# A novel mutation in the NAGLU gene associated with Sanfilippo syndrome type B (mucopolysaccharidosis III B)

**DOI:** 10.1002/ccr3.1521

**Published:** 2018-04-14

**Authors:** Dineshani Hettiarachchi, Nilaksha Nethikumara, Bamunu Arachchi Pathiranage Sajeewani Pathirana, Kalum Weththasigha, Weerabaddana Dilshani Niluka Dissanayake, Vajira H. W. Dissanayake

**Affiliations:** ^1^ Human Genetics Unit Faculty of Medicine University of Colombo Colombo Sri Lanka; ^2^ Sirimawo Bandaranayaka Specialised Children's Hospital Peradeniya Sri Lanka

**Keywords:** Lysosomal storage disease, mucopolysaccharidosis, *N*‐alpha‐acetylglucosaminidase, novel mutation, Sanfilippo syndrome

## Abstract

Homozygous or compound heterozygous mutation in the gene encoding *N*‐alpha‐acetylglucosaminidase (NAGLU) on chromosome 17q21 results in Sanfilippo B, resulting in excess accumulation of intralysosomal glycosaminoglycans (mucopolysaccharides) in various tissues. We wish to report a novel homozygous variant in a child with features of Sanfilippo syndrome B.

## Graphical abstract

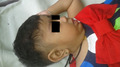

## Introduction

Sanfilippo syndrome is a lysosomal storage disease caused by a deficiency or the absence of four different enzymes that is required for the degradation of heparan sulfate glycosaminoglycans (GAG) found abundantly in the extracellular matrix. Each enzyme deficiency segregates the disease as follows: deficiency of *N*‐sulfoglucosamine sulfohydrolase due to a mutation of its gene on chromosome 17q25 results in Sanfilippo A, homozygous or compound heterozygous mutation in the gene encoding *N*‐alpha‐acetylglucosaminidase (NAGLU) on chromosome 17q21 results in Sanfilippo B, mutations in the HGSNAT gene encoding heparan acetyl‐CoA:alpha‐glucosaminide *N*‐acetyltransferase, on chromosome 8p11, leads to Sanfilippo C, and finally a mutation in the gene encoding *N*‐acetylglucosamine‐6‐sulfatase on chromosome 12q14 results in Sanfilippo D 1, 2. Above mutations result in accumulation of excessive intralysosomal glycosaminoglycans (mucopolysaccharides) in various tissues, causing distended lysosomes to accumulate in the cell and interfere with cell function, and these patients have an increased urinary excretion of GAG and show signs of deterioration of physical and mental status characterized by severe central nervous system degeneration with mild somatic involvement manifesting as intellectual disability, dementia, and a shortened lifespan of 20–30 years 3, 4, 5. Here, we wish to report a novel homozygous variant in a child with features of Sanfilippo syndrome B.

## Case Report

**Figure 1:**
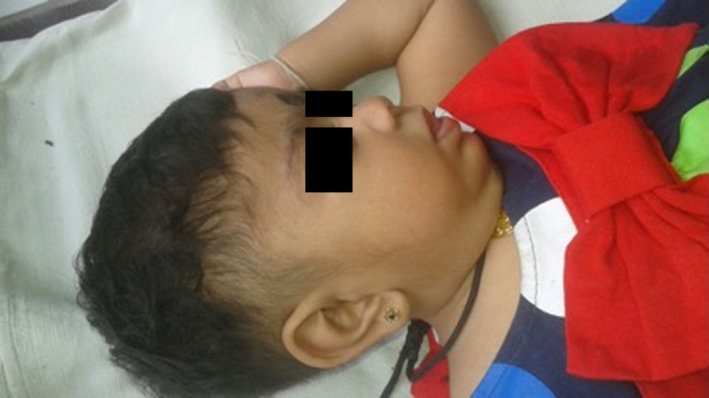
Lateral view of the proband.

**Figure 2:**
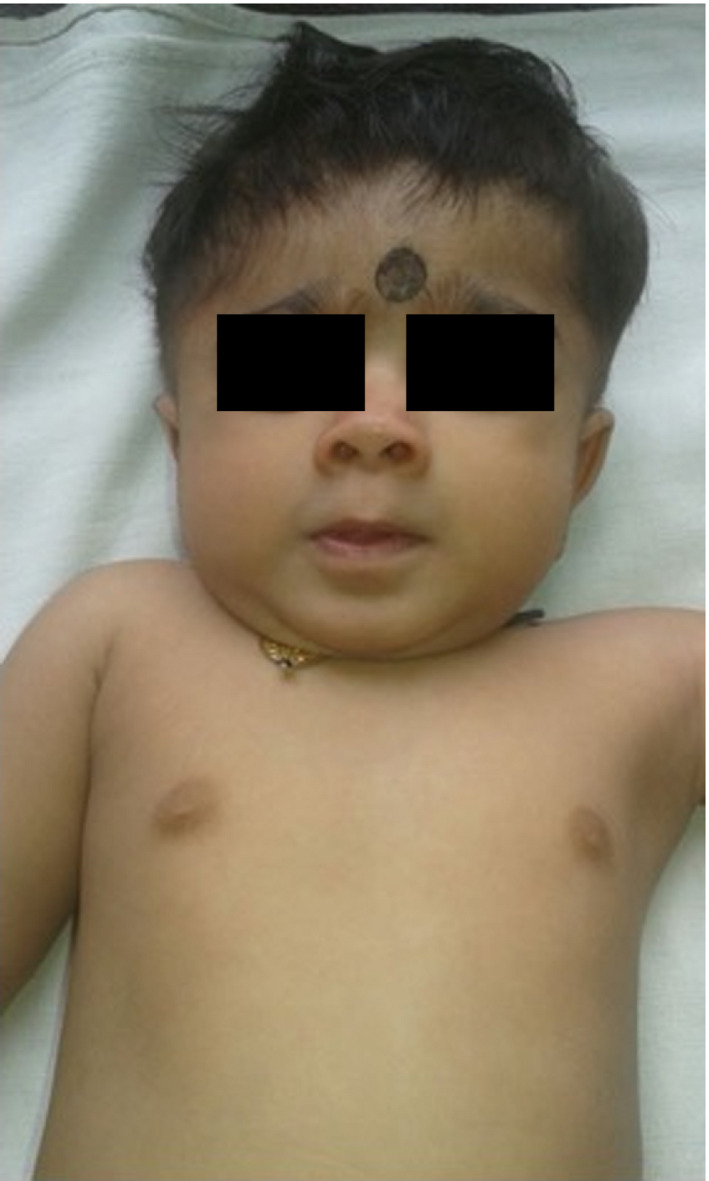
Frontal view of the proband.

The patient of Sri Lankan origin was born after 40 weeks of gestation by normal vaginal delivery. She is the 4th child of an otherwise healthy consanguineous parents. Her first sibling (sister) was diagnosed with Sanfilippo syndrome type B (mucopolysaccharidosis IIIB) and succumbed to illness at the age of 13 years. Her second sibling, a brother, was delivered at 28 weeks of gestation and died soon after birth. Her third sibling (sister) is currently 4 years of age and shows no signs of the diseases and is deemed healthy. The proband had a healthy birthweight of 3.2 kg a length of 50 cm and microcephaly (less than 3 SD below the mean) she cried immediately after birth and reached the required milestones till the age of 6 months after which there was progressive loss of head control and failure to thrive accompanied by global developmental delay and developmental regression characterized by generalized hypotonia, and on further examination, there were mild coarse facies (Figs. 1 and 2). Her investigations showed marked elevation in *α*‐*N*‐acetylglucosaminidase levels, laryngomalacia, pseudoesotropia, plagiocephaly, left pulmonary artery origin stenosis, bilaterally small SVC, osmium secundum atrial septal defect (OS‐ASD), and a normal karyotype (46XX). She also suffered from frequent upper respiratory infections. After obtaining the informed consent of the parents, whole‐exome sequencing was performed.

## Methods

### Whole‐exome sequencing

Whole‐exome sequencing (WES) of the proband's DNA was performed in line with the SureSelect^®^ Human All Exon V6 kit on an Illumina^®^ HiSeq 4000 Next Generation Sequencer. The DNA sequence was compared with the UCSC hg19 reference sequence. All reportable sequence variants were confirmed by visual inspection of the alignment.

### Bioinformatics analysis

Sequenced data were analyzed using an in‐house variant calling annotation pipeline. Paired‐end sequencing data were mapped to the GrCh37 human reference sequence using BWA‐mem algorithm. The resultant sam file was converted to bam, sorted and indexed using Sam Tools. Deduping of reads was performed using Picard Tools. Deduped Bam was realigned around indels and recalibrated using the Genome Analysis Tool Kit (GATK). Variant discovery was performed using GATK Haplotype Caller. VCF file was annotated using SNP‐eff with Refseq, dbSNP, 1000 Genomes, Exome Variant Server, Exome Aggregation Consortium, phastCons100way, ClinVar, and locus‐specific databases. In silico functional prediction was performed using Mutation Taster, SIFT, PolyPhen2, and Provean. Benign variants that were not resided in genes that are known to be causing Sanfilippo Syndrome were filtered out using a virtual gene panel. Retained variants were further scrutinized for their functional impact on the protein, availability in public databases, and the level of conservation in the resided region.

## Results

**Figure 3:**
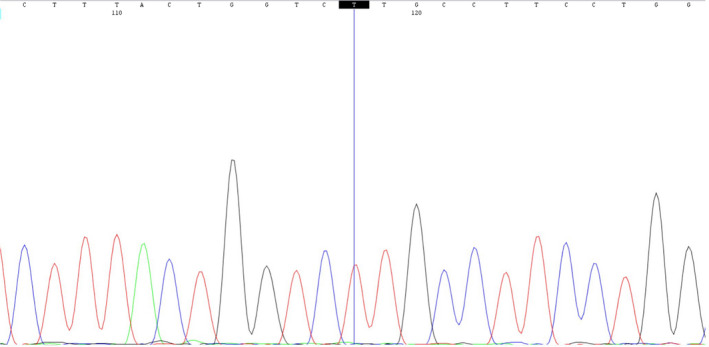
Sanger sequence chromatogram showing a novel homozygous missense mutation in exon 3 of the *N*‐acetyl‐alpha‐glucosaminidase (NAGLU) gene, NM_000263.3: c.587C>T [NP_000254.2: p.Pro196Leu] causing Sanfilippo syndrome.

A novel missense mutation in exon 3 of the *N*‐acetyl‐alpha‐glucosaminidase (NAGLU) gene, NM_000263.3: c.587C>T [NP_000254.2: p.Pro196Leu] causing Sanfilippo syndrome, was detected (read depth: 73x). The patient was a homozygote for the mutation. Mutation was also seen in the Sanger sequence chromatogram (Fig. 3). P196L was a novel mutation and therefore is absent in population genetic databases and clinical databases. It results in a nonconservative amino acid substitution, which impacts the physiochemical nature of the protein. This mutation resides in a highly conserved region among different species throughout the evolution (phastCons100way_vertebrate score = 1). In silico prediction tools universally concluded that p.Pro196Leu has a deleterious effect (Table 1) on protein structure and function.

**Table 1:** Results of in silico mutation prediction analysis

Algorithm	Prediction	Score
PolyPhen2^a^	Probably damaging (HumDiv model)	1.000
PolyPhen2^a^	Probably damaging (HumVar model)	0.994
Provean^b^	Deleterious	−9.72
SIFT^c^	Damaging	0.000
Mutation Taster^d^	Disease causing	0.999

a http://genetics.bwh.harvard.edu/pph2; deleterious threshold >0.5.

b http://provean.jcvi.org/index.php; score threshold is −2.5 for binary classification.

c http://sift.jcvi.org/www/SIFT_chr_coords_submit.html threshold <0.05.

d http://www.mutationtaster.org; Scores range from 0.0 to 1.0.

## Discussion

Sanfilippo syndrome type B (mucopolysaccharidosis III B) is a lysosomal storage disease caused by a homozygous or compound heterozygous mutation in the gene *N*‐alpha‐acetylglucosaminidase (NAGLU) on chromosome 17q21. Mutations in NAGLU protein causes a rare neurological disease and data analyzed using in vitro enzyme activity from a population of 165 missense mutations extracted from the ExAC exome database showed a unique feature in this variant in particular, as with other monogenic disease‐related mutations structural analysis showed a large fraction operated by destabilizing the three‐dimensional structure of the protein 6. In this study, we identified a novel NAGLU variant in a patient with a clinical suspicion of Sanfilippo syndrome type B. The patient showed the following features associated with the disease: neurological deterioration, progressive slowing mental development, frequent upper respiratory infections, mild coarse facies, and high *α*‐*N*‐acetylglucosaminidase levels. These findings are consistent with the diagnosis, genetic analysis identified a novel missense mutation in exon 3 of the *N*‐acetyl‐alpha‐glucosaminidase (NAGLU) gene, NM_000263.3: c.587C>T [NP_000254.2: p.Pro196Leu], which could explain this phenotype.

Although the exact pathogenesis is unknown, it is thought that the accumulation of heparan sulfate, which is a proteoglycan that binds to many ligands modulating numerous cellular pathways, leads to the neurological dysfunction associated with MPS III. Children with this disease have severe neurological and behavioral symptoms, leading to death in the first 20–30 years of life 7, 8. MPS III has no known cure, and enzyme replacement therapy has been found successful in treating some types of MPS with extensive somatic involvement 9, 10. Engraftment of corrected neural stem cells has also shown promising results in mice by reducing the lysosomal storage of heparin sulfate. These findings show that autologous cells can be used as a system to deliver the missing enzyme 11, 12. Given the devastating nature of the illness and the lack of approved treatment options for patients with MPS IIIB, current supportive care is palliative for behavioral problems, sleep disturbances, seizures, and other complications, and does not address the root cause of MPS IIIB or stop disease progression 13.

In our patient, the P196L was a novel mutation and therefore is absent in population genetic databases and clinical databases. It results in a nonconservative amino acid substitution, which impacts the physiochemical nature of the protein We can conclude that all in silico tools predict a deleterious impact on protein function hence giving rise to the phenotypic features of MPS III.

The following study was conducted following Ethical Approval (EC‐16‐179) from the Ethics Review Committee, Faculty of Medicine – University of Colombo.

## Authorship

DH, WDND, and VHWD were the clinicians looking after the patient. BAPSP and KW performed laboratory testing. NN performed bioinformatics analysis. DH wrote the first draft of the manuscript with contributions from all. All authors reviewed, modified and approved the final version of the manuscript.

## Conflict of Interest

Authors declare no conflict of interests.
